# 2-Methyl­propan-2-aminium 2-(meth­oxy­carbon­yl)benzoate

**DOI:** 10.1107/S1600536811037688

**Published:** 2011-09-30

**Authors:** Jian Li

**Affiliations:** a Department of Chemistry and Chemical Engineering, Weifang University, Weifang 261061, People’s Republic of China

## Abstract

In the title compound, C_4_H_12_N^+^·C_9_H_7_O_4_^−^, two C atoms  and the N atom of the cation lie on a mirror plane, while all the atoms of the anion are disordered about a mirror plane. In the crystal, N—H⋯O hydrogen bonds link the components into chains along [010]. In the anion, the mean planes of the methoxycarbonyl and carboxylate groups form dihedral angles of 83.0 (2) and 83.2 (2)°, respectively, with the aromatic ring.

## Related literature

For the applications of phthalimides and *N*-substituted phthalimides, see: Lima *et al.* (2002 ▶). For related structures, see: Li (2011 ▶); Liang (2011 ▶).
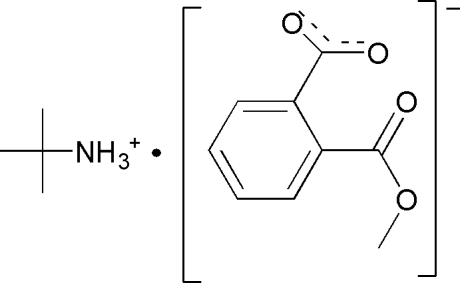


## Experimental

### 

#### Crystal data

C_4_H_12_N^+^·C_9_H_7_O_4_^−^
*M* *_r_* = 253.29
Monoclinic, 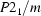

*a* = 9.2939 (8) Å
*b* = 7.0159 (6) Å
*c* = 10.5536 (11) Å
β = 103.322 (1)°
*V* = 669.63 (11) Å^3^
*Z* = 2
Mo *K*α radiation
μ = 0.09 mm^−1^
*T* = 298 K
0.49 × 0.43 × 0.32 mm

#### Data collection

Bruker SMART CCD diffractometer
Absorption correction: multi-scan (*SADABS*; Bruker, 1997 ▶) *T*_min_ = 0.956, *T*_max_ = 0.971
4353 measured reflections
1797 independent reflections
1137 reflections with *I* > 2σ(*I*)
*R*_int_ = 0.021

#### Refinement

*R*[*F* ^2^ > 2σ(*F* ^2^)] = 0.047
*wR*(*F* ^2^) = 0.137
*S* = 1.03
1797 reflections
152 parameters
14 restraints
H atoms treated by a mixture of independent and constrained refinement
Δρ_max_ = 0.16 e Å^−3^
Δρ_min_ = −0.17 e Å^−3^

### 

Data collection: *SMART* (Bruker, 1997 ▶); cell refinement: *SAINT* (Bruker, 1997 ▶); data reduction: *SAINT*; program(s) used to solve structure: *SHELXS97* (Sheldrick, 2008 ▶); program(s) used to refine structure: *SHELXL97* (Sheldrick, 2008 ▶); molecular graphics: *SHELXTL* (Sheldrick, 2008 ▶) and *PLATON* (Spek, 2009 ▶); software used to prepare material for publication: *SHELXTL*.

## Supplementary Material

Crystal structure: contains datablock(s) global, I. DOI: 10.1107/S1600536811037688/lh5313sup1.cif

Structure factors: contains datablock(s) I. DOI: 10.1107/S1600536811037688/lh5313Isup2.hkl

Supplementary material file. DOI: 10.1107/S1600536811037688/lh5313Isup3.cml

Additional supplementary materials:  crystallographic information; 3D view; checkCIF report

## Figures and Tables

**Table 1:** Hydrogen-bond geometry (Å, °)

*D*—H⋯*A*	*D*—H	H⋯*A*	*D*⋯*A*	*D*—H⋯*A*
N1—H2*N*⋯O4	0.925 (18)	1.749 (19)	2.674 (3)	178.3 (17)
N1—H2*N*⋯O3^i^	0.925 (18)	2.042 (18)	2.926 (3)	159.4 (16)
N1—H1*N*⋯O3^ii^	0.92 (3)	1.96 (2)	2.825 (3)	156 (1)
N1—H1*N*⋯O3^iii^	0.92 (3)	1.96 (2)	2.825 (3)	156 (1)

Symmetry codes: (i) 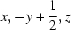; (ii) 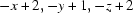; (iii) 
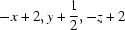
.

**Fig. 1.**
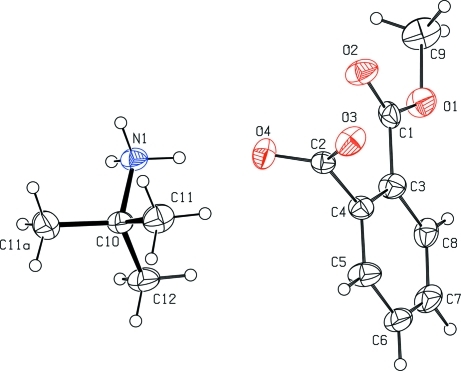
The asymmetric unit of (I), drawn with 30% probability ellipsoids. The disorder is not shown (symmetry code (a): x, -y+3/2, z).

**Fig. 2.**
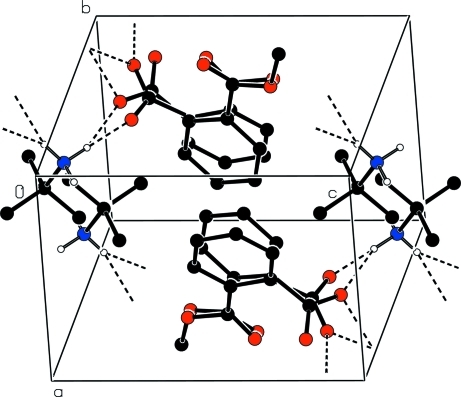
Part of the crystal structure of the title compound with hydrogen bonds shown as dashed lines.

## supplementary crystallographic information

### Comment

Phthalimides and N-substituted phthalimides are animportant class of compounds because of their interesting biological activities (Lima *et al.*, 2002). Tert-butylaminium 2-(methoxycarbonyl)benzoate is an intermediate in the preparation of N-substituted phthalimides. The crystal structures of propan-1-aminium 3,4,5,6-tetrabromo-2-(methoxycarbonyl)benzoate *N*,*N*-dimethylformamide monosolvate (Li, 2011) and butane-1,4-diaminium bis[3,4,5,6-tetrachloro-2-(methoxycarbonyl)benzoate] (Liang, 2011) have already been reported. In this paper, the structure of the title compound is reported. The asymmetric unit of the title compound, 2-methylpropan-2-aminium 2-(methoxycarbonyl)benzoate, (I), is shown in Fig. 1. Atoms C11 and C11a of the cation lie symmetrically on a mirror plane (C10C12N1) while all the atoms of the anion are disordered over a mirror plane. In the crystal, N—H···O hydrogen bonds link the components into one-dimensional chains along [010].

### Experimental

A mixture of phthalic anhydride (1.52 g, 0.01 mol) and methanol (15 ml) was refluxed for 30 min. Then *tert*-butylamine (0.73 g, 0.01 mol) was added to the above solution and mixed for 30 min at room temperature. The solution was kept at room temperature for 5 d. Natural evaporation gave colourless single crystals of the title compound, suitable for X-ray analysis.

### Refinement

H atoms bonded to C atoms were placed in calculated positions and refined in a riding-model approximation with C—H = 0.93-0.96 Å and with *U*_iso_(H) = 1.2*U*_eq_(C) or 1.5*U*_eq_(C_methyl_). H atoms bonded to N were refined independently with isotropic displacement parameters.

### Crystal data

**Table:**

C_4_H_12_N^+^·C_9_H_7_O_4_^−^	*F*(000) = 272
*M**_r_* = 253.29	*D*_x_ = 1.256 Mg m^−^^3^
Monoclinic, *P*2_1_/*m*	Mo *K*α radiation, λ = 0.71073 Å
Hall symbol: -P 2yb	Cell parameters from 1362 reflections
*a* = 9.2939 (8) Å	θ = 2.6–25.8°
*b* = 7.0159 (6) Å	µ = 0.09 mm^−^^1^
*c* = 10.5536 (11) Å	*T* = 298 K
β = 103.322 (1)°	Block, colorless
*V* = 669.63 (11) Å^3^	0.49 × 0.43 × 0.32 mm
*Z* = 2	

### Data collection

**Table:**

Bruker SMART CCD diffractometer	1797 independent reflections
Radiation source: fine-focus sealed tube	1137 reflections with *I* > 2σ(*I*)
graphite	*R*_int_ = 0.021
φ and ω scans	θ_max_ = 28.4°, θ_min_ = 2.6°
Absorption correction: multi-scan (*SADABS*; Bruker, 1997)	*h* = −12→11
*T*_min_ = 0.956, *T*_max_ = 0.971	*k* = −9→9
4353 measured reflections	*l* = −6→14

### Refinement

**Table:**

Refinement on *F*^2^	Primary atom site location: structure-invariant direct methods
Least-squares matrix: full	Secondary atom site location: difference Fourier map
*R*[*F*^2^ > 2σ(*F*^2^)] = 0.047	Hydrogen site location: inferred from neighbouring sites
*wR*(*F*^2^) = 0.137	H atoms treated by a mixture of independent and constrained refinement
*S* = 1.03	*w* = 1/[σ^2^(*F*_o_^2^) + (0.0609*P*)^2^ + 0.1129*P*] where *P* = (*F*_o_^2^ + 2*F*_c_^2^)/3
1797 reflections	(Δ/σ)_max_ < 0.001
152 parameters	Δρ_max_ = 0.16 e Å^−^^3^
14 restraints	Δρ_min_ = −0.17 e Å^−^^3^

### Special details

**Table:**

Geometry. All esds (except the esd in the dihedral angle between two l.s. planes) are estimated using the full covariance matrix. The cell esds are taken into account individually in the estimation of esds in distances, angles and torsion angles; correlations between esds in cell parameters are only used when they are defined by crystal symmetry. An approximate (isotropic) treatment of cell esds is used for estimating esds involving l.s. planes.
Refinement. Refinement of F^2^ against ALL reflections. The weighted R-factor wR and goodness of fit S are based on F^2^, conventional R-factors R are based on F, with F set to zero for negative F^2^. The threshold expression of F^2^ > 2sigma(F^2^) is used only for calculating R-factors(gt) etc. and is not relevant to the choice of reflections for refinement. R-factors based on F^2^ are statistically about twice as large as those based on F, and R- factors based on ALL data will be even larger.

### Fractional atomic coordinates and isotropic or equivalent isotropic displacement parameters (Å^2^)

**Table:**

	*x*	*y*	*z*	*U*_iso_*/*U*_eq_	Occ. (<1)
O1	0.8688 (2)	0.2267 (9)	0.39796 (18)	0.0599 (11)	0.50
O2	0.9747 (2)	0.2711 (10)	0.60624 (19)	0.0596 (10)	0.50
O3	0.8655 (3)	0.1366 (4)	0.8604 (3)	0.0541 (7)	0.50
O4	0.8413 (3)	0.4448 (4)	0.8097 (3)	0.0544 (7)	0.50
C1	0.8655 (3)	0.2433 (7)	0.5222 (2)	0.0440 (7)	0.50
C2	0.8117 (3)	0.2730 (9)	0.7889 (2)	0.0366 (9)	0.50
C3	0.7136 (3)	0.2236 (5)	0.5430 (2)	0.0470 (9)	0.50
C4	0.6905 (3)	0.2377 (6)	0.6689 (2)	0.0407 (6)	0.50
C5	0.5479 (3)	0.2184 (7)	0.6863 (3)	0.0574 (13)	0.50
H5A	0.5315	0.2273	0.7698	0.069*	0.50
C6	0.4291 (3)	0.1862 (4)	0.5818 (3)	0.0547 (9)	0.50
H6A	0.3342	0.1727	0.5955	0.066*	0.50
C7	0.4520 (4)	0.1743 (4)	0.4584 (3)	0.0566 (9)	0.50
H7A	0.3725	0.1551	0.3879	0.068*	0.50
C8	0.5922 (3)	0.1906 (3)	0.4389 (3)	0.0483 (8)	0.50
H8A	0.6070	0.1797	0.3551	0.058*	0.50
C9	1.0136 (3)	0.2437 (12)	0.3673 (3)	0.0716 (11)	0.5
H9A	1.0025	0.2370	0.2747	0.107*	0.50
H9B	1.0762	0.1417	0.4083	0.107*	0.50
H9C	1.0573	0.3637	0.3989	0.107*	0.50
N1	0.87003 (19)	0.7500	0.96516 (19)	0.0428 (5)	
C10	0.7457 (2)	0.7500	1.0343 (2)	0.0417 (5)	
C11	0.76193 (19)	0.5723 (3)	1.11787 (18)	0.0606 (5)	
H11A	0.7587	0.4617	1.0637	0.091*	
H11B	0.6826	0.5666	1.1621	0.091*	
H11C	0.8547	0.5761	1.1808	0.091*	
C12	0.6025 (2)	0.7500	0.9300 (2)	0.0601 (7)	
H12A	0.5986	0.8615	0.8766	0.090*	0.50
H12B	0.5200	0.7504	0.9706	0.090*	
H12C	0.5983	0.6381	0.8769	0.090*	0.50
H2N	0.8597 (18)	0.643 (2)	0.9127 (18)	0.060 (5)*	
H1N	0.960 (3)	0.7500	1.024 (3)	0.060 (7)*	

### Atomic displacement parameters (Å^2^)

**Table:**

	*U*^11^	*U*^22^	*U*^33^	*U*^12^	*U*^13^	*U*^23^
O1	0.0603 (11)	0.080 (3)	0.0415 (9)	−0.011 (2)	0.0155 (8)	−0.014 (2)
O2	0.0444 (9)	0.088 (3)	0.0444 (9)	−0.012 (2)	0.0060 (7)	−0.006 (2)
O3	0.0524 (14)	0.0535 (17)	0.0458 (16)	−0.0050 (13)	−0.0106 (12)	0.0165 (13)
O4	0.0625 (17)	0.0448 (16)	0.0515 (17)	−0.0075 (12)	0.0042 (13)	−0.0091 (12)
C1	0.0504 (12)	0.0467 (15)	0.0338 (12)	0.006 (5)	0.0073 (10)	−0.014 (4)
C2	0.0340 (9)	0.042 (2)	0.0338 (10)	0.0011 (13)	0.0088 (8)	0.0018 (14)
C3	0.0417 (12)	0.059 (3)	0.0369 (12)	0.0012 (18)	0.0015 (9)	−0.0022 (19)
C4	0.0364 (10)	0.0458 (15)	0.0364 (11)	0.005 (3)	0.0014 (8)	0.001 (3)
C5	0.0396 (12)	0.086 (4)	0.0453 (13)	−0.001 (2)	0.0064 (10)	0.001 (2)
C6	0.0357 (14)	0.056 (2)	0.068 (2)	−0.0041 (11)	0.0018 (14)	−0.0015 (14)
C7	0.0465 (16)	0.059 (2)	0.0528 (18)	−0.0036 (13)	−0.0126 (14)	−0.0040 (14)
C8	0.0556 (17)	0.046 (2)	0.0366 (14)	−0.0011 (12)	−0.0028 (12)	−0.0038 (11)
C9	0.0723 (18)	0.086 (3)	0.0611 (19)	−0.015 (5)	0.0253 (15)	0.032 (5)
N1	0.0340 (9)	0.0543 (12)	0.0378 (10)	0.000	0.0037 (7)	0.000
C10	0.0327 (10)	0.0499 (12)	0.0418 (11)	0.000	0.0069 (8)	0.000
C11	0.0581 (10)	0.0617 (11)	0.0619 (10)	−0.0050 (8)	0.0137 (8)	0.0120 (9)
C12	0.0361 (11)	0.0756 (17)	0.0636 (15)	0.000	0.0010 (11)	0.000

### Geometric parameters (Å, °)

**Table:**

O1—C1	1.323 (3)	C8—H8A	0.9300
O1—C9	1.460 (3)	C9—H9A	0.9600
O2—C1	1.200 (3)	C9—H9B	0.9600
O3—C2	1.249 (6)	C9—H9C	0.9600
O4—C2	1.244 (7)	N1—C10	1.501 (3)
C1—C3	1.484 (4)	N1—H2N	0.925 (18)
C2—C4	1.509 (3)	N1—H1N	0.92 (3)
C3—C4	1.397 (4)	C10—C11^i^	1.515 (2)
C3—C8	1.400 (4)	C10—C11	1.515 (2)
C4—C5	1.386 (4)	C10—C12	1.519 (3)
C5—C6	1.388 (4)	C11—H11A	0.9600
C5—H5A	0.9300	C11—H11B	0.9600
C6—C7	1.370 (5)	C11—H11C	0.9600
C6—H6A	0.9300	C12—H12A	0.9600
C7—C8	1.369 (5)	C12—H12B	0.9600
C7—H7A	0.9300	C12—H12C	0.9600
			
C1—O1—C9	116.4 (2)	O1—C9—H9B	109.5
O2—C1—O1	122.5 (2)	H9A—C9—H9B	109.5
O2—C1—C3	125.3 (2)	O1—C9—H9C	109.5
O1—C1—C3	112.2 (2)	H9A—C9—H9C	109.5
O4—C2—O3	126.5 (4)	H9B—C9—H9C	109.5
O4—C2—C4	113.5 (3)	C10—N1—H2N	107.8 (11)
O3—C2—C4	119.9 (3)	C10—N1—H1N	110.8 (15)
C4—C3—C8	119.0 (3)	H2N—N1—H1N	110.9 (13)
C4—C3—C1	119.6 (2)	N1—C10—C11^i^	107.38 (11)
C8—C3—C1	121.4 (2)	N1—C10—C11	107.38 (11)
C5—C4—C3	118.7 (2)	C11^i^—C10—C11	110.77 (19)
C5—C4—C2	117.2 (2)	N1—C10—C12	106.99 (18)
C3—C4—C2	124.1 (2)	C11^i^—C10—C12	112.01 (11)
C4—C5—C6	121.4 (3)	C11—C10—C12	112.01 (11)
C4—C5—H5A	119.3	C10—C11—H11A	109.5
C6—C5—H5A	119.3	C10—C11—H11B	109.5
C7—C6—C5	119.7 (3)	H11A—C11—H11B	109.5
C7—C6—H6A	120.1	C10—C11—H11C	109.5
C5—C6—H6A	120.1	H11A—C11—H11C	109.5
C8—C7—C6	120.0 (3)	H11B—C11—H11C	109.5
C8—C7—H7A	120.0	C10—C12—H12A	109.5
C6—C7—H7A	120.0	C10—C12—H12B	109.5
C7—C8—C3	121.2 (3)	H12A—C12—H12B	109.5
C7—C8—H8A	119.4	C10—C12—H12C	109.5
C3—C8—H8A	119.4	H12A—C12—H12C	109.5
O1—C9—H9A	109.5	H12B—C12—H12C	109.5

Symmetry codes: (i) *x*, −*y*+3/2, *z*.

### Hydrogen-bond geometry (Å, °)

**Table:**

*D*—H···*A*	*D*—H	H···*A*	*D*···*A*	*D*—H···*A*
N1—H2N···O4	0.925 (18)	1.749 (19)	2.674 (3)	178.3 (17)
N1—H2N···O3^ii^	0.925 (18)	2.042 (18)	2.926 (3)	159.4 (16)
N1—H1N···O3^iii^	0.92 (3)	1.96 (2)	2.825 (3)	156.(1)
N1—H1N···O3^iv^	0.92 (3)	1.96 (2)	2.825 (3)	156.(1)

Symmetry codes: (ii) *x*, −*y*+1/2, *z*; (iii) −*x*+2, −*y*+1, −*z*+2; (iv) −*x*+2, *y*+1/2, −*z*+2.
